# Marine records reveal multiple phases of Toba’s last volcanic activity

**DOI:** 10.1038/s41598-023-37999-w

**Published:** 2023-07-18

**Authors:** B. Caron, G. Del Manzo, B. Villemant, A. Bartolini, E. Moreno, A. Le Friant, F. Bassinot, F. Baudin, A. Alves

**Affiliations:** 1 Institut des Sciences de la Terre de Paris, UMR 7193, Sorbonne Université, CNRS-INSU, 75252 Paris cedex 05, France; 2 Université Paris Cité, Institut de Physique du Globe de Paris, CNRS, UMR 7154, 75005 Paris, France; 3 Centre de Recherche en Paléontologie-Paris, UMR 7207, Muséum National d’Histoire Naturelle, CNRS, Sorbonne Université, 75005 Paris, France; 4 Laboratoire d’Océanographie et du Climat: Expérimentations et Approches Numériques, UMR 7159 CNRS, IRD, Sorbonne Université/MNHN/IPSL, 75252 Paris cedex 05, France; 5 Laboratoire des Sciences du Climat et de l’Environnement LSCE/IPSL, UMR CEA-CNRS-UVSQ 8212, 91191 Gif-sur-Yvette, France

**Keywords:** Volcanology, Climate change, Palaeoclimate, Climate-change adaptation, Geochemistry, Volcanology, Palaeontology

## Abstract

The Indonesian Young Toba Tuff (YTT), classically dated around 74 ka BP, is considered as a short-lived explosive cataclysmic super-eruption. The huge amounts of ash and SO_2_ emitted are likely to have triggered a volcanic winter which accelerated the transition to the last glaciation, and may have induced a human genetic bottleneck. However, the global climatic impact of the YTT or its duration are hotly debated. The present work offers a new interpretation of the Toba volcanic complex eruptive history. Analysing the BAR94-25 marine core proximal to the Toba volcanic center and combining it with high-resolution tephrostratigraphy and δ^18^O stratigraphy, we show that the Toba complex produced a volcanic succession that consists of at least 17 distinct layers of tephra and cryptotephra. Textural and geochemical analyses show that the tephra layers can be divided in 3 main successive volcanic activity phases (VAP1 to VAP3) over a period of ~ 50 kyr. The main volcanic activity phase, VAP2, including the YTT, is likely composed of 6 eruptive events in an interval whose total duration is ~ 10 ka. Thus, we suggest that the eruptive model of the Toba volcano must be revised as the duration of the Toba volcanic activity was much longer than suggested by previous studies. The implications of re-estimating the emission rate and the dispersion of ashes and SO_2_ include global environmental reconstitutions, climate change modelling and possibly human migration and evolution.

## Introduction

The potential impact on climate of the Young Toba Tuff (YTT) explosive super-eruption, which occurred ~ 74 ka ago in Sumatra^1–5^, is the subject of a lively debate^6–8^. The main question is whether a single eruption of exceptional magnitude^1^ could accelerate the entry of the Earth into the last glacial period^4^. Assuming that the YTT eruption was one pulse, *i.e.* within an age window of ~ 74–75 ka, many studies have questioned the hypothesis that this eruption had any durable impact on climate during the transition to glacial Marine Isotope Stage 4 (MIS4)^3,9,10^. The importance of its environmental consequences is also questioned on the basis of estimated SO_2_ emission budget^3^ and of archeological and paleobotanical records^11–13^.

**Figure 1:**
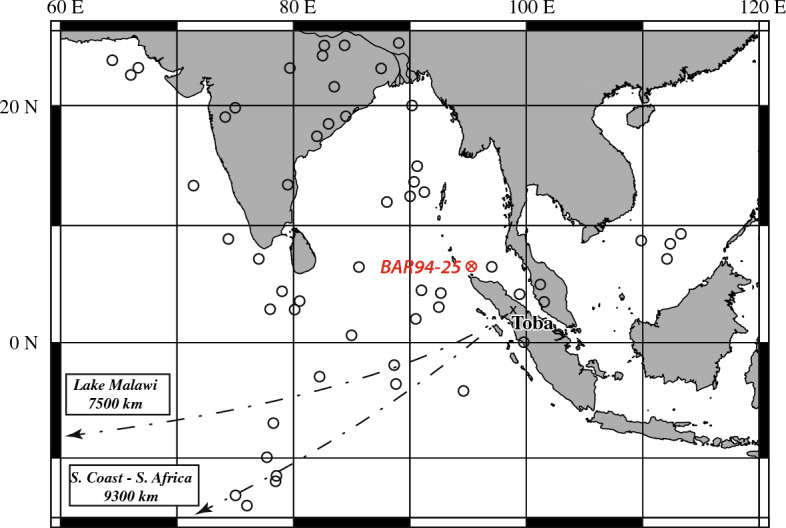
Location of the marine BAR94-25 (red circle crossed) core and other marine and on-land deposits^1–3,11,25^ (black circle). BAR94-25 core has been recovered by 1558 m water depth at 6°26.09′ N and 95°19.5′ E during the BARRAT cruise on the Baruna Jaya I ship, 1994^19^). Cross: Toba Caldera on Sumatra island. Black circles: locations where Toba deposits have been recognized on the basis of clasts textures and major element compositions: Bay of Bengal and Indian Subcontinent^1,22^, Central Indian Basin^2,23^, South China Sea [^14^, S10], Arabian Sea^25^, South and Central Africa^11,12^.

The exceptionally large magnitude of the Toba’s volcanic activity is indisputable. The Toba’s caldera is the largest known Quaternary caldera (3500 km^2^) and the emitted magma volume is estimated > 2800–5300 km^3^ (dense rock equivalent^2,7^). A tephra layer assigned to the YTT can be identified over almost the entire Indian Ocean, in South of China Sea and as far South as Central Africa (Fig. 1)^9,11,12,14,15^. However, the eruptive story of the YTT remains poorly constrained^3^_._ The most popular eruptive scenario suggests that the eruption was a single event of short duration (dozens of days)^1,16^, which classifies the YTT as a cataclysmic eruption of extreme intensity. This scenario has been established in the 1970s on the basis of graded bedding and sedimentation rate estimation of deep-sea tephra layers^1,16^. However, the discovery of numerous sulphate peaks in polar ice cores dated over a period of 2000 years around 74 ka fueled the hypothesis that more than one Toba eruption^17,18^ may have occurred over a relatively short period. The Toba origin of these sulphate peaks remains unfortunately unconfirmed because no Toba tephra have been identified in the polar ice cores.

To clarify the Toba’s eruptive history at the last interglacial/glacial transition, we carried out a high-resolution tephrostratigraphic study of marine core BAR94-25^19^, located 100 km north-west of Sumatra, close to the Toba eruptive center (Fig. 1, *see Fig.* S1*of Suppl Info*). Marine sediment cores proximal to volcanic centers are powerful archives to reconstruct past eruptive histories because they provide better preserved tephra layers than on-land deposits, easily affected by weathering, erosion and by younger volcanic deposit coverage^20^.

Major and trace element compositions of hundreds of individual glass shards and micro-pumices have been determined as different geochemical populations emerged, discriminating their magmatic source and evolution. New δ^18^O measurements complete the previously published data set^19^ that improve the chronostratigraphy of core BAR94-25 which was obtained by aligning the planktonic δ^18^O record to the astronomically-tuned, Low Latitude Stack (LLS)^21^ (*see the detailed explanation in the* Suppl. Info).

## Results

**Figure 2:**
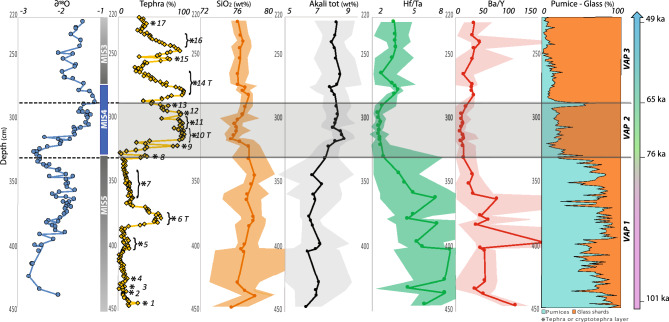
The volcanic record in BAR94-25 marine core (clasts abundances and textures, geochemistry). Chronology and correlation with Marine Isotope Stages. Orbitally-tuned chronostratigraphy was developed based on planktic foraminifer *G. ruber* δ^18^O data. Tephrostratigraphy: Tephra%: clast fraction in decarbonated sediment (cm scale); stars indicate the identified 17 volcanic units. *T* are tephra layers visible to the naked eye, other were considered as cryptotephra. Pumice-Glass fraction is the ratio: number of pumice clasts (green) over the total number of pumices + glass shards (orange). Geochemistry: Total alkali contents, Hf/Ta and Ba/Y ratios. Color area (orange, grey, green and red) represent the range of the geochemical composition. δ^18^O data^19^. Age model is from Bassinot^21^. Paleoclimatic proxy: Marine Isotope Stages (MIS5 is interglacial warm period; MIS4 is a glacial cold period; MIS3 is an enigmatic “temperate” period [S11]). Names of Volcanic Activity Periods (VAP 1, 2 and 3) are defined on the basis of textural and geochemical characteristics and of chronology (see text).

We studied the core from 447 to 219 cm depth, spanning the time interval c. 100–50 ka^19,21^ based on planktic foraminifer δ^18^O stratigraphy (Fig. 2). The ages presented in this document are potentially subject to significant uncertainties, although difficult to quantify. In the literature, the few attempts to estimate the uncertainties associated with astronomically derived dating procedures have concluded that they could be on the order of a few thousand years (~ 2–5 ka). However, in the rest of the article, for the sake of simplicity and ease of describing the figures, the ages of the events will not be rounded (example: the age of the bottom of the VAP3 event is given at 48 ka, although we could have rounded it to 50 ka).

**Figure 3:**
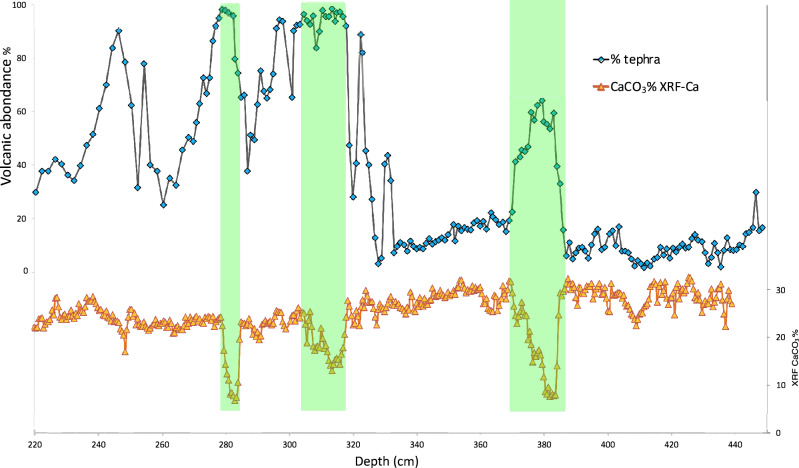
Comparison between high-resolution record of CaCO_3_ content and high-resolution tephrostratigraphy of core BAR94-25 between 220 and 440 cm depth. Green zones underline 3 tephra layers, with a low CaCO_3_ proportion in bulk sediment.

**Figure 4:**
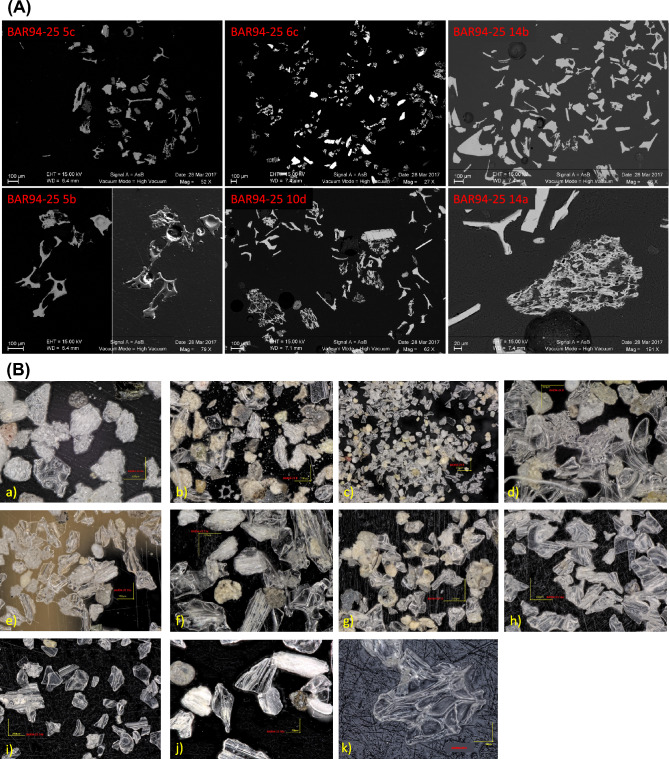
(**A**) Images SEM of various tephra and cryptotephra layer from the BAR94-25 core. (**B**) Images from a Keyence VHX 7000 numerical microscope of tephra and cryptotephra layers from the BAR94-25 core. (**a**) Layer 6a; (**b**) layer 8; (**c**, **d**) layer 9; (**e**) layer 10a; (**f)** layer 11a; (**g**) layer 12; (**h**) layer 14a; (**i**) layer 14b; (**j**) layer 16a; (**k**) sample detail of layer 9. (High resolution images are available with numerical version).

Throughout the studied interval sediments are fairly homogeneous olive oozes with a rather invariant CaCO_3_ content (20–30%), with the exception of three tephra layers, where sediments show a marked decrease in calcium carbonate content. This is due to the relatively fast deposition of tephra: each of these tephra or crypto-tephra layers likely induced a rapid and short-lasting increase in sedimentation rate as the volcanic events behind provided large fluxes of material compared to the slow hemipelagic sedimentation. Figure 3 shows the comparison between high-resolution record of CaCO_3_ content and volcanic ash content of core BAR94-25. Tephra were sampled to the cm scale that includes the so-called YTT ash layer (~ 74 ka), observed at 318 cm^19^ downcore and an older ash layer located at 382.5 cm^19^. Ca XRF data along the core show 3 zones with very low values (279.5–284.5/311.5–318.5 and 377–385 cm depth respectively) that confirm visible naked eye tephra layer (Fig. 3). Overall, thirty-five volcanic deposits (tephra and crypto-tephra) were identified and gathered to 17 distinct volcanic events (Fig. 2, visible naked eye tephra layers are noted *T, see photo of BAR94-25 sections Fig.* S1*Suppl. Info*). The tephra layers are composed of glass shards and micro-pumice up to several hundred µm in size (Fig. 4). There is a sharp transition between the pumice-rich (70% of the decarbonated material) and the glass shard-rich (85% of the decarbonated material) tephra units at 332 cm. Micro-pumice fragments (ca. 100 µm) are highly vesiculated, aphyric, with a glassy groundmass. Glass shards are large (> 200 µm), aphyric with few rounded bubbles. Glass shards generally display curved shapes from the fragmentation and quenching of large bubbles (> > 200 µm). SEM images and numerical microscope observations of tephra layers are available Fig. 4a and b. All fragments are rhyolitic in composition (76–78 wt% SiO_2_, Table 1 and all data table in Appendix). Trace element compositions are typical of highly evolved rhyolitic melts. Glass shards collected in tephra layers above and below 332 cm display subtle yet systematic differences in composition: above 332 cm, K_2_O contents are higher (mean 4.8 ± 0.02 wt% against 3.72 ± 0.43), and MgO and CaO contents are lower (respectively: mean 0.07 ± 0.02 wt% against 0.19 ± 0.06 and 0.79 ± 0.03 wt% against 1.21 ± 0.21 wt%). The color area in Fig. 2 (orange, grey, green and red) represents the range of the geochemical composition and shows the heterogeneity or the homogeneity of tephra layers composition.

**Table 1:** Geochemical compositions (mean, standard deviation and RSD) values of major elements (and alkali) in Wt% of the 17 tephra layers.

	Depth	Name	Nb	SiO_2_	TiO_2_	Al_2_O_3_	Fe_2_O_3_ T	MnO	MgO	CaO	Na_2_O	K_2_O	P_2_O_5_	F	Cl	Alkali
VAP3	225.5	17	17	76.54	0.11	12.93	0.90	0.08	0.08	0.80	3.52	4.80	0.06	0.15	0.13	8.32
			*SD*	*0.39*	*0.07*	*0.32*	*0.17*	*0.05*	*0.03*	*0.10*	*0.27*	*0.49*	*0.04*	*0.09*	*0.03*	*0.38*
			*RSD*	*0.51*	*68.93*	*2.49*	*19.18*	*66.16*	*31.84*	*13.09*	*7.76*	*10.20*	*62.31*	*59.31*	*22.25*	*4.59*
	235.5	16.c	15	76.79	0.11	12.93	0.84	0.06	0.08	0.77	3.64	4.57	0.04	0.17	0.13	8.21
			*SD*	*0.38*	*0.05*	*0.28*	*0.13*	*0.05*	*0.02*	*0.06*	*0.19*	*0.28*	*0.03*	*0.09*	*0.02*	*0.30*
			*RSD*	*0.50*	*48.20*	*2.20*	*15.75*	*86.82*	*26.56*	*7.99*	*5.19*	*6.10*	*78.58*	*54.28*	*16.18*	*3.70*
	240.5	16.b	14	77.01	0.12	12.94	0.80	0.08	0.08	0.79	3.67	4.31	0.05	0.11	0.13	7.98
			*SD*	*0.42*	*0.07*	*0.23*	*0.15*	*0.04*	*0.03*	*0.19*	*0.16*	*0.27*	*0.04*	*0.07*	*0.03*	*0.23*
			*RSD*	*0.54*	*60.21*	*1.80*	*18.83*	*56.76*	*30.99*	*23.91*	*4.33*	*6.21*	*67.63*	*65.23*	*22.95*	*2.91*
	245.5	16.a	15	76.72	0.13	12.97	0.83	0.07	0.09	0.77	3.70	4.50	0.05	0.12	0.15	8.19
			*SD*	*0.56*	*0.05*	*0.25*	*0.12*	*0.05*	*0.02*	*0.06*	*0.38*	*0.22*	*0.04*	*0.08*	*0.02*	*0.50*
			*RSD*	*0.73*	*38.52*	*1.92*	*14.47*	*62.65*	*22.54*	*7.36*	*10.19*	*4.79*	*73.94*	*62.75*	*15.47*	*6.08*
	253.5	15	16	76.71	0.09	12.82	0.91	0.07	0.09	0.81	3.66	4.62	0.06	0.09	0.13	8.29
			*SD*	*0.45*	*0.05*	*0.32*	*0.14*	*0.04*	*0.04*	*0.16*	*0.25*	*0.48*	*0.05*	*0.05*	*0.04*	*0.45*
			*RSD*	*0.58*	*57.71*	*2.51*	*15.04*	*58.29*	*50.22*	*19.45*	*6.74*	*10.30*	*78.57*	*61.18*	*27.47*	*5.40*
	265.5	14.e	17	76.49	0.09	12.82	0.94	0.07	0.07	0.75	3.33	5.23	0.04	0.14	0.16	8.56
			*SD*	*0.47*	*0.06*	*0.26*	*0.13*	*0.06*	*0.03*	*0.09*	*0.26*	*0.37*	*0.04*	*0.10*	*0.02*	*0.25*
			*RSD*	*0.62*	*65.98*	*2.01*	*13.45*	*76.44*	*42.36*	*12.09*	*7.89*	*7.10*	*89.36*	*73.81*	*14.83*	*2.92*
	273.5	14.d	23	76.54	0.13	12.89	0.88	0.10	0.09	0.83	3.75	4.59	0.05	0.17	0.13	8.33
			*SD*	*0.38*	*0.09*	*0.24*	*0.11*	*0.06*	*0.04*	*0.14*	*0.34*	*0.50*	*0.04*	*0.10*	*0.03*	*0.36*
			*RSD*	*0.50*	*66.39*	*1.90*	*12.53*	*60.03*	*44.12*	*16.52*	*9.01*	*10.84*	*78.98*	*62.25*	*21.99*	*4.31*
	275.5	14.c	11	77.24	0.12	12.77	0.84	0.08	0.09	0.81	3.63	4.25	0.07	0.06	0.14	7.88
			*SD*	*0.61*	*0.08*	*0.22*	*0.11*	*0.03*	*0.04*	*0.08*	*0.72*	*0.15*	*0.04*	*0.05*	*0.01*	*0.71*
			*RSD*	*0.79*	*68.22*	*1.70*	*13.24*	*35.84*	*48.67*	*10.47*	*19.77*	*3.59*	*57.74*	*84.24*	*4.86*	*8.96*
	278.5	14.b	8	76.99	0.12	12.82	0.88	0.06	0.12	0.78	3.78	4.24	0.04	0.10	0.15	8.02
			*SD*	*0.44*	*0.06*	*0.27*	*0.14*	*0.05*	*0.05*	*0.05*	*0.49*	*0.21*	*-*	*0.10*	*0.02*	*0.55*
			*RSD*	*0.57*	*53.19*	*2.07*	*15.77*	*75.22*	*43.41*	*6.54*	*13.04*	*4.92*	*-*	*102.10*	*12.85*	*6.92*
	281.5	14.a	8	77.55	0.16	12.24	0.85	0.05	0.08	0.73	3.93	4.28	0.02	0.04	0.15	8.21
			*SD*	*0.34*	*0.10*	*0.21*	*0.07*	*0.04*	*0.02*	*0.02*	*0.29*	*0.21*	*0.01*	*0.03*	*0.02*	*0.31*
			*RSD*	*0.44*	*64.93*	*1.70*	*8.23*	*81.34*	*27.25*	*2.21*	*7.37*	*4.85*	*59.63*	*62.46*	*10.49*	*3.79*
VAP2	290.5	13	6	77.21	0.07	12.30	0.97	0.04	0.06	0.80	3.52	4.79	0.07	0.05	0.15	8.32
			*SD*	*0.39*	*0.05*	*0.56*	*0.18*	*0.04*	*0.02*	*0.06*	*0.24*	*0.50*	*0.05*	*0.03*	*0.02*	*0.29*
			*RSD*	*0.50*	*61.83*	*4.54*	*18.44*	*86.20*	*40.46*	*7.20*	*6.88*	*10.42*	*60.23*	*67.62*	*10.72*	*3.53*
	296.5	12	7	76.97	0.08	12.32	1.02	0.11	0.05	0.83	3.33	5.07	0.06	0.06	0.18	8.39
			*SD*	*0.31*	*0.05*	*0.33*	*0.17*	*0.04*	*0.04*	*0.23*	*0.27*	*0.18*	*0.04*	*0.03*	*0.03*	*0.20*
			*RSD*	*0.41*	*63.04*	*2.65*	*16.57*	*34.67*	*81.09*	*27.98*	*8.24*	*3.51*	*74.42*	*48.90*	*17.01*	*2.38*
	300.5	11.c	13	76.51	0.08	13.16	0.95	0.07	0.05	0.73	3.17	5.11	0.04	0.07	0.14	8.28
			*SD*	*0.86*	*0.06*	*0.82*	*0.15*	*0.04*	*0.03*	*0.09*	*0.26*	*0.17*	*0.03*	*0.04*	*0.01*	*0.23*
			*RSD*	*1.13*	*75.75*	*6.22*	*15.53*	*59.74*	*55.00*	*11.84*	*8.14*	*3.36*	*69.45*	*53.54*	*9.49*	*2.75*
	303.5	11.b	10	76.22	0.12	13.31	1.00	0.08	0.06	0.77	3.05	5.17	0.04	0.07	0.18	8.22
			*SD*	*0.47*	*0.08*	*0.30*	*0.08*	*0.05*	*0.02*	*0.08*	*0.41*	*0.28*	*0.03*	*0.04*	*0.07*	*0.37*
			*RSD*	*0.62*	*65.22*	*2.27*	*7.67*	*56.45*	*34.07*	*10.62*	*13.56*	*5.38*	*63.85*	*61.91*	*40.83*	*4.45*
	306.5	11.a	6	76.42	0.06	13.37	0.98	0.05	0.05	0.77	3.06	5.02	0.03	0.05	0.14	8.09
			*SD*	*0.36*	*0.07*	*0.47*	*0.13*	*0.03*	*0.02*	*0.08*	*0.27*	*0.14*	*0.04*	*0.03*	*0.02*	*0.34*
			*RSD*	*0.48*	*116.93*	*3.50*	*13.71*	*63.03*	*37.49*	*10.52*	*8.81*	*2.70*	*152.58*	*53.79*	*13.39*	*4.18*
	309.5	10.d	8	76.38	0.07	12.84	0.94	0.11	0.06	0.78	3.59	5.01	0.10	0.07	0.15	8.59
			*SD*	*0.64*	*0.05*	*0.43*	*0.12*	*0.09*	*0.03*	*0.08*	*0.17*	*0.22*	*0.07*	*0.05*	*0.04*	*0.17*
			*RSD*	*0.84*	*72.02*	*3.36*	*13.15*	*81.81*	*54.21*	*10.55*	*4.80*	*4.44*	*64.21*	*69.69*	*24.32*	*2.03*
	312.5	10.c	13	76.15	0.09	12.86	1.06	0.12	0.07	0.81	3.49	5.20	0.10	0.10	0.14	8.69
			*SD*	*0.43*	*0.06*	*0.27*	*0.15*	*0.07*	*0.03*	*0.13*	*0.19*	*0.26*	*0.03*	*0.08*	*0.04*	*0.19*
			*RSD*	*0.57*	*68.57*	*2.07*	*14.50*	*62.12*	*40.80*	*16.20*	*5.49*	*4.97*	*27.74*	*78.14*	*27.93*	*2.16*
	315.5	10.b	8	75.90	0.08	13.11	0.92	0.05	0.06	0.84	3.68	5.17	0.13	0.06	0.14	8.85
			*SD*	*0.43*	*0.06*	*0.37*	*0.12*	*0.06*	*0.03*	*0.09*	*0.17*	*0.29*	*0.07*	*0.04*	*0.03*	*0.37*
			*RSD*	*0.57*	*71.49*	*2.82*	*13.52*	*103.89*	*41.32*	*10.53*	*4.53*	*5.58*	*56.11*	*68.84*	*18.45*	*4.22*
	317.5	10.a	11	76.70	0.08	12.96	0.96	0.05	0.05	0.78	3.42	4.79	0.04	0.04	0.14	8.22
			*SD*	*0.80*	*0.08*	*0.66*	*0.10*	*0.03*	*0.03*	*0.11*	*0.16*	*0.34*	*0.05*	*0.03*	*0.03*	*0.41*
			*RSD*	*1.04*	*93.24*	*5.08*	*9.93*	*62.58*	*54.79*	*13.80*	*4.70*	*7.04*	*120.80*	*84.94*	*18.32*	*5.01*
	321.5	9	17	77.56	0.09	12.23	0.93	0.10	0.05	0.79	3.17	4.62	0.03	0.61	0.14	7.79
			*SD*	*0.51*	*0.05*	*0.34*	*0.14*	*0.06*	*0.02*	*0.07*	*0.20*	*0.22*	*0.02*	*0.41*	*0.02*	*0.25*
			*RSD*	*0.65*	*61.37*	*2.80*	*14.93*	*63.28*	*38.54*	*8.23*	*6.37*	*4.74*	*79.85*	*66.84*	*14.29*	*3.26*
	330.5	8	24	77.57	0.07	12.52	0.98	0.08	0.06	0.77	2.93	4.63	0.03	0.57	0.14	7.56
			*SD*	*0.71*	*0.05*	*0.32*	*0.13*	*0.05*	*0.02*	*0.09*	*0.36*	*0.18*	*0.02*	*0.37*	*0.02*	*0.38*
			*RSD*	*0.91*	*68.64*	*2.53*	*13.38*	*60.48*	*35.45*	*11.60*	*12.26*	*3.99*	*73.90*	*65.80*	*17.29*	*5.06*
VAP1	343.5	7.d	6	78.12	0.12	12.57	0.86	0.06	0.17	1.10	3.04	3.67	0.05	0.39	0.12	6.71
			*SD*	*0.97*	*0.06*	*0.69*	*0.37*	*0.04*	*0.10*	*0.36*	*0.81*	*0.87*	*0.03*	*0.42*	*0.05*	*0.65*
			*RSD*	*1.24*	*50.81*	*5.48*	*43.22*	*64.69*	*59.97*	*32.59*	*26.63*	*23.67*	*71.73*	*108.47*	*42.93*	*9.66*
	350.5	7.c	4	77.81	0.11	12.12	1.09	0.06	0.11	0.92	2.98	4.36	0.03	0.38	0.13	7.34
			*SD*	*0.53*	*0.13*	*0.59*	*0.15*	*0.05*	*0.09*	*0.20*	*0.44*	*0.39*	*0.03*	*0.31*	*0.04*	*0.78*
			*RSD*	*0.69*	*112.82*	*4.88*	*13.81*	*78.28*	*77.26*	*21.29*	*14.66*	*8.91*	*92.62*	*80.97*	*31.55*	*10.57*
	358.5	7.b	10	77.42	0.16	12.88	0.99	0.04	0.13	1.01	2.57	4.22	0.04	0.85	0.14	6.79
			*SD*	*1.17*	*0.08*	*0.42*	*0.20*	*0.03*	*0.07*	*0.16*	*0.56*	*0.51*	*0.03*	*0.65*	*0.03*	*0.78*
			*RSD*	*1.51*	*50.46*	*3.28*	*20.25*	*66.05*	*54.84*	*16.11*	*21.59*	*12.16*	*82.54*	*76.75*	*24.36*	*11.55*
	362.5	7.a	7	77.37	0.20	12.74	1.10	0.07	0.18	1.10	2.79	4.11	0.04	0.36	0.13	6.90
			*SD*	*1.35*	*0.13*	*0.59*	*0.27*	*0.05*	*0.11*	*0.36*	*0.29*	*0.60*	*0.03*	*0.19*	*0.03*	*0.62*
			*RSD*	*1.75*	*65.14*	*4.61*	*24.54*	*83.16*	*60.59*	*32.82*	*10.30*	*14.49*	*81.75*	*53.61*	*26.60*	*8.93*
	375.5	6.c	12	78.00	0.17	12.37	1.00	0.05	0.20	1.20	2.98	3.52	0.04	0.54	0.19	6.50
			*SD*	*0.59*	*0.03*	*0.31*	*0.13*	*0.04*	*0.02*	*0.11*	*0.30*	*0.14*	*0.02*	*0.42*	*0.11*	*0.37*
			*RSD*	*0.76*	*21.18*	*2.50*	*13.35*	*72.44*	*12.35*	*9.50*	*10.02*	*4.03*	*61.93*	*78.20*	*55.55*	*5.70*
	378.5	6.b	7	77.98	0.17	12.50	0.97	0.05	0.20	1.18	2.91	3.70	0.04	0.33	0.17	6.61
			*SD*	*0.86*	*0.09*	*0.47*	*0.17*	*0.05*	*0.04*	*0.15*	*0.28*	*0.08*	*0.03*	*0.18*	*0.02*	*0.21*
			*RSD*	*1.10*	*52.64*	*3.79*	*17.91*	*102.13*	*21.61*	*12.73*	*9.75*	*2.30*	*66.71*	*54.21*	*12.33*	*3.15*
	382.5	6.a	4	77.59	0.19	12.60	0.98	0.07	0.17	1.10	2.91	3.82	0.04	0.61	0.16	6.74
			*SD*	*0.93*	*0.10*	*0.31*	*0.15*	*0.06*	*0.07*	*0.15*	*0.19*	*0.32*	*0.05*	*0.54*	*0.04*	*0.42*
			*RSD*	*1.20*	*50.81*	*2.47*	*14.96*	*86.03*	*39.45*	*13.24*	*6.48*	*8.45*	*139.54*	*88.05*	*23.06*	*6.20*
	396.5	5.c	7	77.19	0.21	12.67	1.04	0.08	0.20	1.20	3.32	3.91	0.15	0.03	0.17	7.23
			*SD*	*0.60*	*0.06*	*0.48*	*0.15*	*0.06*	*0.03*	*0.16*	*0.45*	*0.62*	*0.06*	*0.01*	*0.05*	*0.65*
			*RSD*	*0.78*	*30.29*	*3.79*	*14.19*	*74.13*	*12.97*	*13.63*	*13.54*	*15.91*	*37.80*	*37.80*	*31.22*	*8.95*
	400.5	5.b	6	76.84	0.20	12.90	1.03	0.11	0.23	1.23	3.04	3.91	0.07	1.08	0.18	6.95
			*SD*	*0.84*	*0.09*	*0.24*	*0.20*	*0.05*	*0.05*	*0.09*	*0.66*	*0.40*	*0.03*	*0.61*	*0.05*	*0.97*
			*RSD*	*1.10*	*46.32*	*1.86*	*19.52*	*46.46*	*21.07*	*7.66*	*21.53*	*10.25*	*44.72*	*56.11*	*26.53*	*13.93*
	402.5	5.a	8	77.12	0.19	12.91	1.18	0.11	0.22	1.49	3.54	2.86	0.05	0.51	0.16	6.39
			*SD*	*3.29*	*0.08*	*1.69*	*0.45*	*0.08*	*0.09*	*0.14*	*0.89*	*0.66*	*0.03*	*0.34*	*0.09*	*1.27*
			*RSD*	*4.26*	*43.00*	*13.12*	*37.71*	*68.03*	*38.19*	*9.44*	*25.09*	*23.28*	*54.01*	*67.73*	*53.95*	*19.86*
	426.5	4	5	76.49	0.18	13.08	1.24	0.13	0.24	1.34	3.49	3.65	0.05	0.03	0.16	7.14
			*SD*	*1.94*	*0.14*	*1.11*	*0.19*	*0.08*	*0.10*	*0.93*	*1.00*	*1.04*	*0.02*	*0.01*	*0.08*	*0.28*
			*RSD*	*2.54*	*79.88*	*8.51*	*15.62*	*57.73*	*42.37*	*69.67*	*28.63*	*28.49*	*50.00*	*32.59*	*49.39*	*3.99*
	432.5	3	7	77.21	0.16	12.63	1.13	0.10	0.19	1.27	3.39	3.55	0.04	0.27	0.14	6.94
			*SD*	*0.78*	*0.18*	*0.84*	*0.28*	*0.04*	*0.12*	*0.38*	*0.38*	*0.64*	*0.04*	*0.13*	*0.05*	*0.45*
			*RSD*	*1.00*	*117.12*	*6.67*	*24.56*	*38.71*	*66.14*	*29.71*	*11.20*	*18.11*	*102.67*	*48.80*	*35.07*	*6.54*
	436.5	2	3	78.16	0.25	12.38	0.83	0.07	0.10	1.03	3.09	3.89	0.03	0.27	0.11	6.97
			*SD*	*1.49*	*0.08*	*0.97*	*0.24*	*0.04*	*0.12*	*0.54*	*0.59*	*0.86*	*0.01*	*0.15*	*0.09*	*0.29*
			*RSD*	*1.90*	*31.36*	*7.84*	*29.21*	*57.86*	*121.37*	*52.66*	*19.23*	*22.18*	*44.78*	*57.74*	*83.72*	*4.14*
	445.5	1	8	75.94	0.21	13.79	1.37	0.11	0.36	1.75	3.36	2.91	0.05	0.03	0.19	6.28
			*SD*	*0.98*	*0.08*	*0.48*	*0.27*	*0.08*	*0.03*	*0.27*	*0.40*	*0.17*	*0.02*	*0.01*	*0.02*	*0.41*
			*RSD*	*1.29*	*39.17*	*3.47*	*19.98*	*71.35*	*7.74*	*15.53*	*11.81*	*5.80*	*35.36*	*39.17*	*11.21*	*6.47*

**Figure 5:**
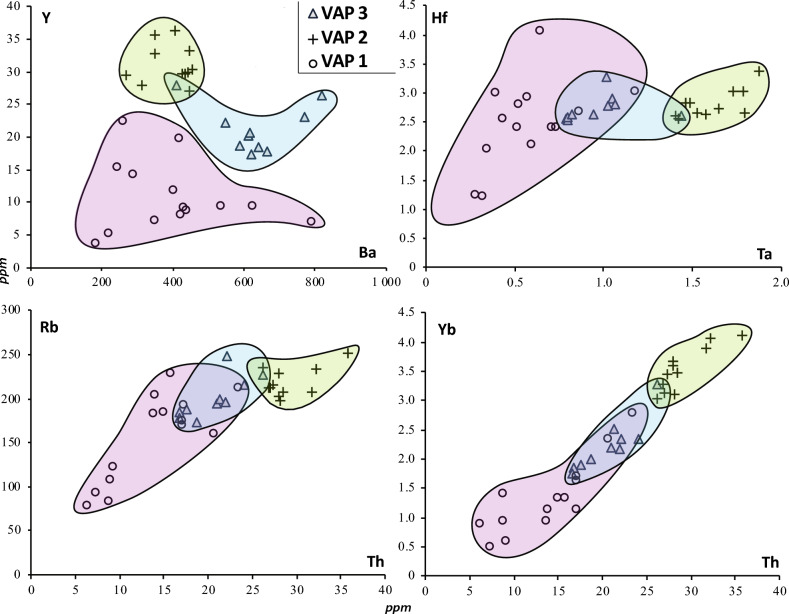
Geochemistry: trace element compositions (Ba, Y, Hf, Ta, Rb, Yb, Th; LA-ICP-MS/MS measurements) of the three main periods: VAP 1, 2 and 3. The VAP 2 products have the most homogeneous composition and correspond to the most differentiated magma. Conversely, the VAP 1 magmas are the most heterogeneous and the less differentiated. All magmas are rhyolitic in composition.

Textural observations of the clasts suggest micro-pumice or glass shard fallouts 600 km far from the eruptive center (Fig. 1). On this basis, along with the geochemical signatures (value and range, Figs. 2 and 5, Tables 1, 2 and all data table in supplementary material), three main explosive eruptive phases (called thereafter Volcanic Activity Phases, VAP) are identified:

**Table 2:** Geochemical compositions (mean, standard deviation and RSD) values of SiO_2_ and Alkali (Na_2_O + K_2_O) in Wt% and trace elements (Rb, Y, Ba, Yb, Hf, Ta and Th) in ppm.

	Depth	Name	*n* =	SiO_2_	Alkali	Hf/Ta	Ba/Y	Rb	Y	Ba	Yb	Hf	Ta	Th
VAP3	225.5	17	17	76.54	8.32	3.02	36.01	216.44	23.10	771.19	2.35	2.95	1.05	24.12
	235.5	16.c	15	76.79	8.21	3.13	32.45	194.51	20.15	613.08	2.19	2.75	0.94	20.97
	240.5	16.b	14	77.01	7.98	3.10	46.42	184.40	17.82	666.48	1.85	2.42	0.79	16.79
	245.5	16.a	15	76.72	8.19	3.17	35.86	178.66	17.44	618.68	1.76	2.52	0.79	16.64
	253.5	15	16	76.71	8.29	2.87	32.22	200.21	22.25	548.26	2.51	2.67	1.06	21.31
	265.5	14.e	17	76.49	8.56	1.88	15.87	226.74	28.05	410.80	3.27	2.57	1.44	26.15
	273.5	14.d	23	76.54	8.33	2.84	32.02	196.69	20.64	616.42	2.18	2.69	1.02	21.92
	275.5	14.c	11	77.24	7.88	3.13	31.17	248.35	26.41	820.69	2.35	3.16	1.01	22.06
	278.5	14.b	8	76.99	8.02	3.36	31.35	173.54	18.76	587.92	2.00	2.66	0.79	18.73
	281.5	14.a	8	77.55	8.21	3.06	34.90	188.05	18.36	639.42	1.90	2.52	0.82	17.48
VAP2	290.5	13	6	77.21	8.32	1.97	19.75	212.16	27.17	447.04	3.14	2.51	1.40	27.06
	296.5	12	7	76.97	8.39	1.61	12.24	251.42	36.29	405.52	4.11	2.98	1.88	35.75
	300.5	11.c	13	76.51	8.28	1.66	12.46	207.52	32.81	350.91	3.90	2.80	1.73	31.67
	303.5	11.b	10	76.22	8.22	1.88	15.15	197.91	29.95	441.31	3.11	2.78	1.49	28.13
	306.5	11.a	6	76.42	8.09	1.66	11.85	236.17	28.04	311.30	3.05	2.29	1.42	26.20
	309.5	10.d	8	76.38	8.59	1.71	9.35	202.24	29.53	267.27	3.59	2.64	1.57	27.96
	312.5	10.c	13	76.15	8.69	1.65	15.12	228.34	33.29	446.06	3.68	2.81	1.79	27.95
	315.5	10.b	8	75.90	8.85	1.78	16.77	208.43	29.67	432.25	3.48	2.84	1.64	28.45
	317.5	10.a	11	76.70	8.22	1.76	11.03	234.58	35.78	350.16	4.07	3.05	1.78	32.25
	321.5	9	17	77.56	7.79	1.85	15.36	216.24	30.42	455.52	3.45	2.83	1.52	27.36
	330.5	8	24	77.57	7.56	2.07	15.67	213.10	29.70	424.49	3.28	2.77	1.46	26.86
VAP1	343.5	7.d	6	78.12	6.71	3.47	32.84	184.80	11.76	402.20	1.32	1.98	0.59	14.93
	350.5	7.c	4	77.81	7.34	3.98	26.48	213.35	22.39	259.74	2.79	2.98	1.17	23.41
	358.5	7.b	10	77.42	6.79	4.69	34.15	169.72	15.34	245.61	1.69	2.43	0.73	17.11
	362.5	7.a	7	77.37	6.90	6.69	78.78	174.68	14.26	287.17	1.64	2.60	0.70	17.11
	375.5	6.c	12	78.00	6.50	4.69	47.96	183.45	7.29	348.09	0.93	2.32	0.51	13.72
	378.5	6.b	7	77.98	6.61	4.23	62.05	92.90	3.71	184.65	0.49	1.21	0.27	7.27
	382.5	6.a	4	77.59	6.74	6.82	40.13	123.41	5.13	218.05	0.61	1.35	0.31	9.15
	396.5	5.c	7	77.19	7.23	5.01	168.05	204.22	8.68	438.65	1.15	2.46	0.52	13.89
	400.5	5.b	6	76.84	6.95	5.51	47.55	228.22	9.29	430.06	1.35	2.86	0.56	15.80
	402.5	5.a	8	77.12	6.39	7.97	55.26	83.08	8.08	421.04	0.93	2.11	0.34	8.76
	426.5	4	5	76.49	7.14	7.51	56.28	107.27	9.30	625.15	1.40	2.93	0.39	8.82
	432.5	3	7	77.21	6.94	4.30	36.86	160.94	19.89	417.40	2.35	2.50	0.86	20.58
	436.5	2	3	78.16	6.97	7.55	53.15	192.43	9.43	537.19	1.13	4.06	0.64	17.14
	445.5	1	8	75.94	6.28	5.72	114.09	78.81	6.93	789.03	0.88	2.44	0.43	6.24

Hf/Ta and Ba/Y ratio of tephra layers are used for the Fig. 2. Depth is in cm.

1. VAP1 from 447 (bottom) to 330 cm (~ 103–76 ka) consists of 7 volcanic events (units 1–7), with pumice fragments generally more abundant than glass shards (mean pumice fraction 70%; range: 24–98%), relatively high MgO (0.19 ± 0.06 wt%) and CaO (1.21 ± 0.21 wt%) contents and relatively low total alkali (6.82 ± 0.31 wt%) and K_2_O contents (3.72 ± 0.43 wt%). Trace element composition of the 7 events is relatively homogeneous with the highly incompatible elements showing the lowest contents, except for Ba. They are characterized by high Hf/Ta ratio (5.6) and very low and slightly variable Ta/Ba, Ba/Y, La/Ba and Yb/Ba ratios.

2. VAP2 from 330 to 290 cm (~ 76–65 ka) consists of 6 volcanic events (units 8 to 13) with high glass shard abundances (mean glass shard fraction 78%; range: 35–94%), high and variable contents in total alkali (7.6–8.9 wt%) and K_2_O (4.6–5.2 wt%). Highly incompatible elements display linear correlations and their large composition ranges indicate large variations in the differentiation degree (Fig. 5, Table 2). They are characterized by a low Hf/Ta ratio (1.8), and high and strongly variable Ta/Ba, Ba/Y, La/Ba and Yb/Ba ratios.

3. VAP3 from 290 to 219 cm (~ 65–48 ka) consist of 4 volcanic events (units 14–17) with abundant glass shards (mean glass shard fraction 88%; range 42–99%). The MgO and CaO contents match the whole range of the magmatic phases 1 and 2, and the alkali contents are intermediate (Na_2_O + K_2_O ~ 8.2%; K_2_O ~ 4.5 wt%). All incompatible trace element ratios (*i.e.* Hf/Ta, Ta/Ba, Ba/Y, La/Ba and Yb/Ba) are constant and low, and close to the highest values of the previous periods, except Hf/Ta which is significantly higher (2.9).

## Discussion

**Figure 6:**
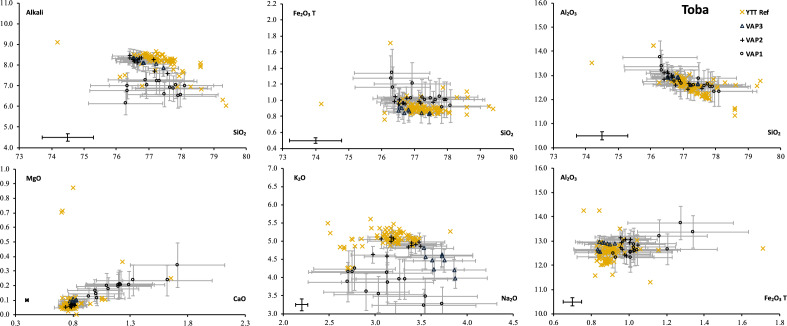
Comparison between data of YTT major element references and our work. Barres on samples (grey) are standard deviation of each element of each tephra layer composition. Barres in down left corner (black) is standard deviation on BHVO-2G standard. The 70 analyses attributed to the YTT eruption were obtained from Gatti [S12].

All tephra found in BAR94-25’s between ~ 100 and 50 ka are linked by their geochemical composition to the activity of Toba^2,3,10,11,22–24^. Tephra from VAP2 and VAP3, covering the time interval ~ 76–48 ka, have the same major element composition (SiO_2_, total alkali, Fe_2_O_3_t, Al_2_O_3_, MgO, CaO) as YTT deposits recorded on-land in Indonesia and in all deep-marine cores^2,3,10,11,22–25^ (see the comparison of literature’s data with this work in Fig. 6). The morphology of volcanic particles (both micro-pumices and glass shards) and specially their large size (Fig. 4a and b) is a characteristic of Toba’s fallout^2^. Considering the adequate distance of BAR94-25 to the volcanic center of the Toba (600 km, Fig. 1), this core likely provides the most complete record of Toba’s activity, in which even the lower intensity eruption phases were recorded, contrary to remote marine cores previously studied that are located thousands of kilometers away^27^.

Different magmatic units are defined on the basis of trace element composition in Toba’s deposits collected on-land and offshore^23,24^. Using Ba, Th, Y, Ta, Hf and Rb, the 17 tephra layers of the BAR94-25 core show 3 major successive volcanic eruptive phases (Figs. 2, 5, 6 and Tables 1 and 2). Although VAP 2 (~ 76–65 ka) and 3 (~ 65–48 ka) have similar trace element compositions as reported for all other YTT deposits^23,24^, some second-order differences allow to distinguish them.

We show that the 74 ka YTT layer^1,3,22^ is included in VAP2 characterized by the most homogeneous glass compositions with the highest differentiation degree and by the highest explosive eruption frequency (Figs. 2, 5, Tables 1 and 2). VAP3 is characterized by less differentiated and slightly more heterogeneous magma compositions and by decreasing explosive eruption frequency (Figs. 2, 5, Tables 1 and 2). Late VAP2 events and VAP3 events correspond chronologically to the late dome forming activity posterior to YTT explosive activity^26^. During VAP1, the Toba volcano complex was mainly active from 90 to 85 ka. An early phase had already been reported in another marine core off western Sumatra^27^. This early phase is now clearly distinguished from subsequent phases by higher pumice abundance and different trace element composition.

Our results show that the pyroclastic material that is related to the Toba volcano complex was not emitted during a single super-eruption event, but rather during a long sequence of discrete volcanic phases (VAP1-VAP3) that peaked in the most massive phase 2 (that includes YTT tephra). Each volcanic phase is composed of several explosive events. Our results are in harmony with the suggestions that the large caldera of Toba collapsed in stages during subsequent volcanic phases^28,29^. Although the age model for the BAR94-25 core is limited in terms of accuracy (see Suppl. Info), our data show that tephra having the same geochemical signature as the main YTT deposit were emitted by at least 6 eruptive events over a total time interval of ~ 10 ka. The sulfate record of Greenland and Antarctic ice cores also suggest the existence of multiple high-frequency volcanic events at that time, but over a shorter period (~ 2 ka) and their study has been limited to the time window around 74 ka^17,30^.

## Conclusive remarks

As can be seen from BAR94-25 marine record, the 6 eruptive events of the Toba eruptive phase 2 took place across the MIS5 and 4 transition (interglacial to glacial with an increase in δ^18^O values) and persisted during MIS4 (δ^18^O maximum; Fig. 2). There is no long rest period in volcanic activity between the onset of VAP2 activity and the onset of glaciation contrary to previous conclusions from low resolution marine cores studies^3^. This reactivates the hypothesis that Toba activity may have facilitated the glaciation onset^4^. Such a hypothesis was discredited because the atmospheric impact of sulfur released from a single eruptive event, even with very large magnitude and intensity, would be buffered in a few years^31^. Recent studies show that the ocean can accommodate on a millennium time-scale the cumulative radiative cooling resulting from repeated volcanic eruptions, implying a more prolonged effect on the climate^32^. A series of large-volume explosive eruptions over several thousand years culminating in one massive eruption may have had a more persistent climate impact than a single short-lived cataclysmic event.

This new scenario for a long-lasting Toba activity at the onset of the last glaciation calls for further studies to estimate the duration, frequency and magnitude of these multiple eruptions (in particular whether stratospheric heights were reached), the associated ash and gas (especially sulpfur and halogens) emissions and their dispersion, in order to better understand the climatic impact of large volcanic systems such as the Toba.

## Supplementary Information

Supplementary Figure S1.

Supplementary Tables.

Supplementary Information 3.

## Data Availability

The datasets used and/or analysed during the current study available from the corresponding author on reasonable request.
