# Supplementary figures and images for: Marine records reveal multiple phases of Toba’s last volcanic activity
Source: Sci Rep. 2023 Jul 18;13:11575. doi: 10.1038/s41598-023-37999-w (PMC10354072; doi:10.1038/s41598-023-37999-w)

a

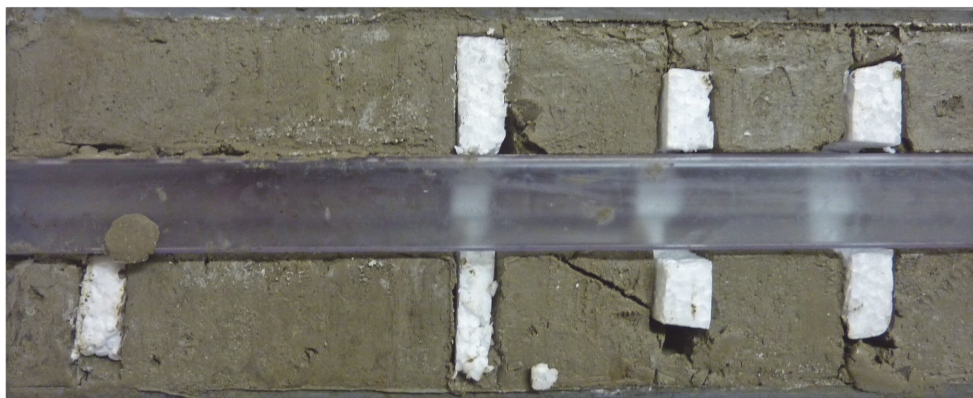

300 305 310 315 320 325

b

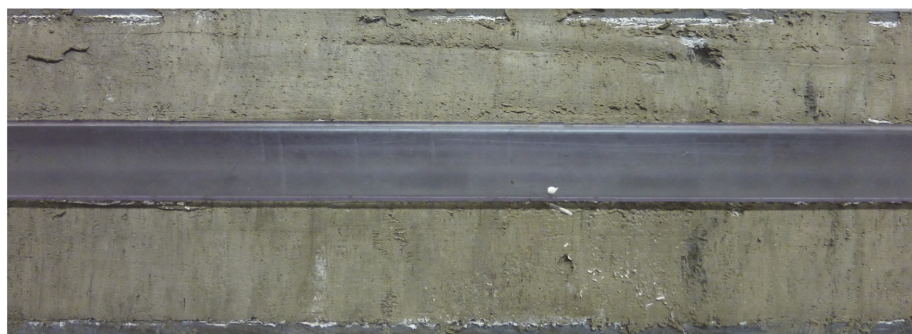

350 355 360 365 370

c

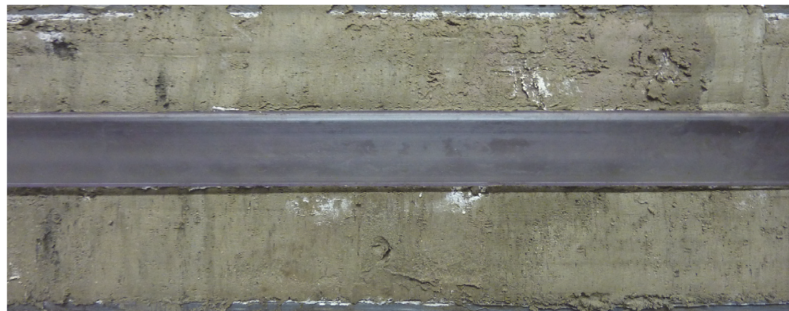

370 375 380 385

Supplement: Supplementary file 1 — Supplementary Figure S1. [file 41598_2023_37999_MOESM1_ESM.pdf]
