# Supplementary material for: Marine records reveal multiple phases of Toba’s last volcanic activity
Source: Sci Rep. 2023 Jul 18;13:11575. doi: 10.1038/s41598-023-37999-w (PMC10354072; doi:10.1038/s41598-023-37999-w)
Supplement: Supplementary file 3 — Supplementary Information 3. [file 41598_2023_37999_MOESM3_ESM.docx]

**Marine records reveal multiple phases of Toba's last volcanic activity.**

***B. Caron^a^, G. Del Manzo^a,b^, B. Villemant^a^, A. Bartolini^c^, E. Moreno^d^, A. Le Friant^b^, F. Bassinot^e^, F. Baudin^a^, A. Alves^c^***

a : Institut des Sciences de la Terre de Paris, UMR 7193, Sorbonne Université, CNRS-INSU,, F-75252 Paris cedex 05, France.

b : Université Paris Cité, Institut de Physique du Globe de Paris, CNRS, UMR 7154, F-75005 Paris, France.

c : Centre de Recherche en Paléontologie - Paris, UMR 7207, Muséum National d’Histoire Naturelle, CNRS, Sorbonne Université,  F-75005 Paris, France.

d : Laboratoire d'Océanographie et du Climat: Expérimentations et approches numériques, UMR 7159 CNRS, IRD, Sorbonne Université/MNHN/IPSL F-75252 Paris cedex 05, France

e : Laboratoire des Sciences du Climat et de l'Environnement LSCE/IPSL, UMR CEA-CNRS-UVSQ 8212 F-91191 Gif-sur-Yvette, France.

***Supplementary Information***

**Methods**

*Oxygen stable isotope analyses and age model*

New δ^18^O measurements complete the previously published data set [19] that improve the sampling spatial resolution to ~2-4 cm over the studied interval (447 to 219 cm depth). Analyses were performed on 10 tests of the surface-dwelling species *Globigerinoides ruber* *s.s.* picked from the 250–315μm size fraction. Tests were ultrasonically cleaned in a methanol bath and then dried naturally in a fume hood. Samples were analyzed with a VG-Optima and a Finnigan IsoPrime mass-spectrometers at the Laboratoire des Sciences du Climat et L'Environnement, Gif-sur-Yvette, France. All results are expressed as δ^18^O in ‰ versus V-PDB with respect from calibrations with NBS 19 and NBS 18 standards. The internal analytical reproducibility determined from replicate measurements of a carbonate standard (Carrara marble UCD-SM92) is ±0.05‰ (1σ).

The chronostratigraphy of core BAR94-25 was obtained by aligning the planktonic δ^18^O record to the astronomically-tuned, Low Latitude Stack (LLS) [21]. The LLS isotope reference curve was chosen here because (i) like BAR94-25 record, it is derived from planktonic foraminifers, and (ii) one of the two isotopic records used to construct the LSS is located in the ocean Tropical Indian [21]. By not fitting the δ^18^O curve to a benthic reference record, we try to limit the potential diachronism issues which affect benthic records during major climate transitions [S1, S2]. Both BAR94-25 and LSS records have well-developed precession-related signals due to the large imprint of this orbital parameter in the tropics, making them easy to align.

Despite the improved resolution of BAR94-25 δ^18^O record, the age model we can derive through tuning to an astronomically calibrated reference curve such LLS has its limitations. Only a few control points can be defined, corresponding to major boundaries (MIS5/MIS4 transition) and climatic optima (*e.g.* MIS5.1, MIS4.0). In addition, due to the chronostratigraphic uncertainties of the LLS target curve itself and the potential errors associated to aligning the BAR94-25 δ^18^O record to this reference target, our age control points have uncertainties of the order of a few thousand years [S3]. Thus, it is beyond the possibility of our age-model to resolve minute changes in sedimentation rates that are associated to specific tephra-rich layers.

Since we did not use the main YTT tephra as an age control point, it can serve to check the robustness of our age model. Based on our δ^18^O-based chronostratigraphic model, the YTT tephra is dated at ~ 72 ka, thus about 2-3 ka younger than the Ar/Ar age of the YTT tephra [2,3,13,24]. This is well within the error bar associated with astronomical tuning [S3].

The deposition of each tephra or crypto-tephra layer likely induced a rapid and short-lasting increase in sedimentation rate as these events provide large fluxes of material compared to the slow hemipelagic sedimentation. The *Fig. 3* shows the comparison between high-resolution record of CaCO_3_ content and volcanic ash content of core BAR94-25 between 220 and 440 cm. CaCO_3_ contents are measured using the X-ray fluorescence (XRF) of Ca. The XRF measurements were done using an Avaatech XRF Core Scanner at the University of Bordeaux EPOC laboratory (France). Every section was scanned at 0.5 cm intervals on u-channels with a counting time of 20 sec and 10 kV. XRF was calibrated using a set of 220 measurements of CaCO_3_ along the whole core (0-600 cm) and determined by reacting 100 mg of the powdered sediment with 1 ml of HCl-8N in carbonate-bomb analyses [S4], using a Mélières-manocalcimeter. The high-resolution CaCO_3_ record was obtained using a linear fit between CaCO_3_ contents and Ca-X Rays intensities (XRF-Ca) measured at equivalent depths: CaCO_3_=1.15 XRF-Ca x10^-4^ + 3.8 (r = 0.75).

The average content in CaCO_3_ in the core is 24-25%. Along the core, 3 zones located at 385, 318.5 and 284.5 cm depth are evidenced by their low Ca-X Rays intensities corresponding to CaCO_3_ contents < 10% (green zone on *Fig. 5* with a thickness of 5, 7 and 8 cm respectively). These intervals correspond to volcanic ash layers visible at the macroscopic level (tephra) and the sedimentation rate is there very strongly increased. However, the content in volcanic glass fragments is not sytematically associated with a significant decrease in CaCO_3_ in the other parts of the core: volcanic ash fragments could only be detected by microscopic observation of the decarbonated fraction above 63µm and were considered as cryptotephra.

The δ^18^O record shows a step-like evolution over some intervals, with values remaining nearly constant between adjacent samples before changing more or less abruptly. The near-constant values could result from contemporaneous foraminifers being buried in rapidly depositing tephra material and thus being smeared over several centimeters of the core. Yet, this interpretation remains just a working hypothesis at this stage. Uncertainties associated to isotopic analyses and the near impossibility of estimating the exact limits of each tephra or crypto-tephra layer due to bioturbation processes, make it impossible to derive a robust volcano-sedimentary model to refine our age model. The age model only provides mean sedimentation rates between the few tuning control points. For this reason, no attempt has been made to estimate the duration of specific tephra or crypto-tephra episodes. Such estimates would be irrelevant given the uncertainties of the astronomically-derived age model and the complexity of the sedimentation processes at play. With the age model in hands, one can only provide estimates regarding the total duration of the three main volcanic activity phases as well as the average frequency of eruptive events within each phase.

The possible remobilization of volcanic deposits within the water column or the post-deposition transport of on-land deposits (in relation with the closeness to the Sumatra shore) are discarded by the δ^18^O stratigraphy that closely fits the reference Low Latitude δ^18^O Stack and by Scanning Electron Microscope (SEM) and X-ray fluorescence (XRF) analyses [19] which do not show any evidence of turbidity, sedimentation gaps or disturbance along the core.

In addition, concerning the marine sediment recording quality, the non-mixing of the geochemical trace compositions strengthens the hypothesis of volcanic fallouts deposits well separated and unmixed (*Fig. 5*).

*Tephra layer analyses:*

The core was sampled at cm-scale between 447 and 219 cm depth (357 samples). Volcanic clasts were identified in decarbonated samples (with MilliQ pure water and HCl 10% v/v) under stereo-microscope. At least 400 particles were counted in every cm of core sample.

We used the conventional tephrostratigraphic method [S5, S6] to characterize crypto-tephra layers and volcanic fragments (361 available analysis of geochemistry composition, peak of volcanic clast abundance) on 35 volcanic layers were studied.

Volcanic clasts were hand-picked and were mounted in epoxy resin beads and polished for systematic Scanning Electron Microscope imaging (*Fig. 4a*, SEM Zeiss Supra 55VP, Sorbonne University, ISTeP facilities), and observed under numerical microscope (*Fig. 4b*, Keyence VHX 7000).

Major and minor elements (Si, Ti, Al, Fe, Mn, Mg, Ca, Na, K, P, F and Cl) analysis were performed using Electron Probe Micro Analyzer (EPMA, SX-FIVE and SX-100 CAMECA instruments; Camparis facilities, Sorbonne University). Analytical conditions were an acceleration voltage of 15 kV, a 4 nA beam current and a 8 μm spot size to prevent significant diffusion of light elements like Na. International USGS (BHVO-2G, BIR-1G, BCR-2G, ATHO) and internal standards (Co5, LGM, LIP) were used to control accuracy and reproducibility of instruments: 10 to 40 tephra fragments were analysed for each identified tephra layer. Replicate standard analyses are reported in supplementary data (Table Excel file ). Comparison with literature values are shown in *Fig. 6*.

Major and trace elements were measured in situ by a Laser Ablation coupled at an Inductively Coupled Plasma Mass Spectrometry (LA-ICP-MS/MS). The Laser Excimer 193nm ArF Teledyne Photon Machine Analyte G2 is coupled to an Agilent ICP-MS/MS 8800 triple quadrupole (ALIPP6 facilities, Sorbonne University [S9, S10]). Tuning was performed on NIST 612 internal standard. Analytical conditions were a frequency of 8 Hz and 2.97 J per cm^2^ of laser fluence with a 40µm beam spot size. Vector gas to carrier the ablated sample to ICP-MS/MS was helium with a flux of 0.5 L.min^-1^. 32 elements were measured simultaneously using 1 or 2 isotopes per element: Si, Ti, Al, Ca, and 28 trace elements (Sc, Co, Ni, Rb, Sr, Y, Zr, Nb, Ba, REE, Hf, Ta, Pb, Th and U). For each run, the analytical consistency was monitored by repeated analysis of international glass references (BCR-2G, BHVO-2G, BIR-1G and ATHO) and internal glass standards (LGM, Co5, LIP). The accuracy and precision of results were calculated from the standard BHVO-2G (Table Excel file). The 8800 ICP-MS/MS was configured in No gas mode and (M/Z)^Q1^=(M/Z)^Q2^ with oxide ratio ^254^[UO]/^238^U <0.015 % and double charged ratio [^44^Ca]^+^/ [^44^Ca]^2+^ <0.2%. The associated error (RSD%) is always lower than 2 % for all trace elements (< 1.5 % for REE). ^238^U/^232^Th≈100% to minimize volatile/refractory fractionation. The element concentration is obtained by normalizing the isotope signal to ^44^Ca signal and multiplying by [Ca] previously measured by EPMA. More details are available in Del Manzo et al., submitted [S10]. Replicate standard analyses are reported in supplementary data (Table Excel file).

***Supplementary Information***

***Figures and Tables:***

**Table** Excel file**:** data of geochemical compositions of tephra and samples. There are the means, the standard deviations and the relative standard deviations of major and trace elements for each tephra and crypto tephra layers.

Excel file: The worksheets are: 1/ figure 2 data, 2/ figure 3 data, 3/ tephra major elements composition, 4/ tephra trace elements composition, 5/ standards major elements compositions, 6/ standards trace elements compositions. Concerning used standards, there are this instrument, the date of the seance, the means, the standard deviations and the relative standard deviations, the reference values and the accuracy of major and trace elements.

**Figure S1:** BAR9425 core: a) interval 300-325 cm, olive-gray fine-grained hemipelagic ooze, intercalated with the tephra layer (318.5-311. 5 cm) of the phase VAP2, barely perceptible, to note that the sediment is free from any disturbance such as bioturbation; b) interval 345-369 cm fairly homogeneous olive-gray fine-grained hemipelagic ooze except for a few levels enriched in organic matter, presence only of cryptotephra (not visible macroscopically); c) interval 365-385 cm, olive-gray fine-grained hemipelagic ooze including the nodular tephra layer (377-385 cm) of the phase VAP1.

**Bibliography appendix**

S1 33 Skinner, L. C., and Shackleton N. J., An Atlantic lead over Pacific deep-water change across Termination I: Implications for the application of the marine isotope stage stratigraphy, *Quaternary Science Reviews*, **24** (5-6), 571–580 (2005). doi:10.1016/j.quascirev.2004.11.008.

S2 34 Lisiecki, L. E., and Stern J. V., Regional and global benthic δ18O stack for the last glacial cycle, *Paleoceanography*, **31**, 1368–1394 (2016). doi:10.1002/2016PA003002.

S3 35 Martinson, D. G., et al., Age dating and the orbital theory of the ice ages: Development of a high-resolution 0 to 300,000-year chronostratigraphy, Quaternary Research., 27 (1), 1–29 (1987). doi.org/10.1016/0033-5894(87)90046-9

S4 36 Müller, G. and Gastner, M., The 'Karbonat-Bombe', a simple device for the determination of carbonate content in sediment, soils, and other materials. Neues Jahrbuch für Mineralogie - Monatshefte, Bremerhaven, *Pangaea* **10**, 466-469. (1971)

S5 37 Caron, B., et al. Late Pleistocene to Holocene tephrostratigraphic record from the Northern Ionian Sea. *Marine Geology* **311–314**, 41–51 (2012).

S6 38 Tanimizu, M., et al., Determination of ultra-low ^236^U/^238^U isotope ratios by tandem quadrupole ICP-MS/MS, *Journal of Analytical Atomic Spectrometry*, **28**,1372 (2013). https://doi.org/10.1039/C3JA50145K

S7 39 Cousin A. et al., SuperCam Calibration Targets on Board the Perseverance Rover: Fabrication and Quantitative Characterization. Spectrochimica Acta Part B: Atomic Spectroscopy ***188*** (2021). https://doi.org/10.1016/j.sab.2021.106341

S8 40 Mauran G., et al., Data pretreatment and multivariate analyses for ochre sourcing: application to Leopard Cave rock art shelter (Erongo, Namibia). *Journal of Archaeological Science* ***35***, (2021) https://doi.org/10.1016/j.jasrep.2020.102757

S9 41 Del Manzo G. et al., High-resolution cryptotephra investigation reveals new eruptive history of Youngest Toba Tuffs (YTT), Sumatra. Submitted.

S10 42 Bühring, C., Sarnthein, M., Leg 184 Shipboard Scientific Party. Toba ash layers in the South China Sea: evidence of contrasting wind directions during eruption ca. 74 kyr. *Geology* **28**, 275–278. (2000).

S11 43 Siddall, M. et al., Marine isotope stage 3 sea level fluctuations: Data synthesis and new outlook. *Reviews Geophysics*,  **46**, (2008), RG4003, doi:[10.1029/2007RG000226](https://doi.org/10.1029/2007RG000226).

S12 44 Gatti, E. et al., Geochemical variability in distal and proximal glass from the Youngest Toba Tuff eruption. *Bulletin of Volcanology*, **76**, (2014), doi.org/10.1007/s00445-014-0859-x
